# (*E*)-5-[(2-Hy­droxy-3-meth­oxy­benzyl­idene)amino]-1,3,4-thia­diazole-2(3*H*)-thione

**DOI:** 10.1107/S1600536811050902

**Published:** 2011-11-30

**Authors:** Hadi Kargar, Reza Kia

**Affiliations:** a Department of Chemistry, Payame Noor University, PO BOX 19395-3697 Tehran, Iran; b X-ray Crystallography Lab., Plasma Physics Research Center, Science and Research Branch, Islamic Azad University, Tehran, Iran and, Department of Chemistry, Science and Research Branch, Islamic Azad University, Tehran, Iran

## Abstract

In the title compound, C_10_H_9_N_3_O_2_S_2_, the dihedral angle between the benzene ring and the five-membered ring is 1.54 (13)°. An intra­molecular O—H⋯N hydrogen bond makes an *S*(6) ring. In the crystal, mol­ecules are linked together through bifurcated N—H⋯(O,O) hydrogen bonds having *R*_1_^2^(5) ring motifs, forming chains along the *b* axis. The crystal structure also features π–π inter­actions, with centroid–centroid distances of 3.699 (3)–3.767 (3) Å.

## Related literature

For standard bond lengths, see: Allen *et al.* (1987 ▶). For hydrogen-bond motifs, see: Bernstein *et al.* (1995 ▶). For the biological versatility of thione ligands, see, for example: Kumar *et al.* (1988 ▶); Yadav *et al.* (1989 ▶). For related structures, see: Zhang (2003 ▶); Kargar *et al.* (2011 ▶, 2011*a* ▶, 2011*b* ▶). 
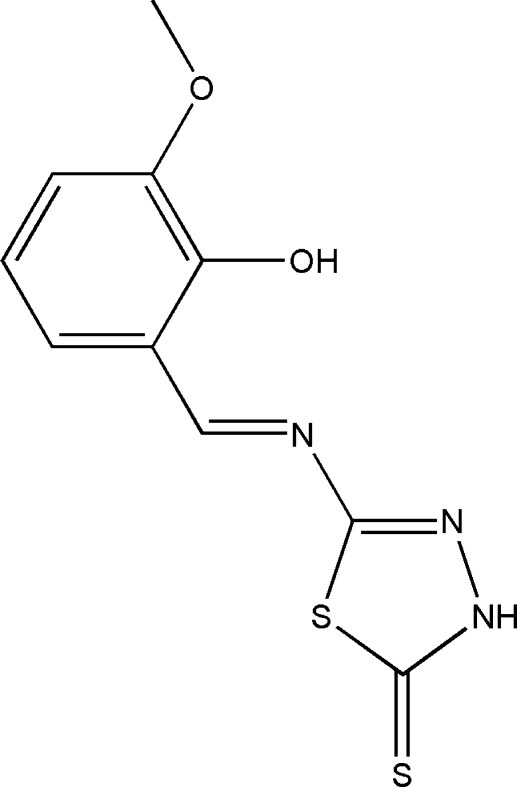


## Experimental

### 

#### Crystal data

C_10_H_9_N_3_O_2_S_2_
*M* *_r_* = 267.32
Monoclinic, 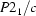

*a* = 7.432 (5) Å
*b* = 14.993 (5) Å
*c* = 10.853 (5) Å
β = 101.738 (5)°
*V* = 1184.0 (10) Å^3^
*Z* = 4
Mo *K*α radiation
μ = 0.44 mm^−1^
*T* = 291 K
0.25 × 0.21 × 0.11 mm

#### Data collection

Stoe IPDS 2T Image Plate diffractometer
Absorption correction: multi-scan [*MULABS* (Blessing, 1995 ▶) in *PLATON* (Spek, 2009 ▶)] *T*_min_ = 0.898, *T*_max_ = 1.000
9012 measured reflections
3147 independent reflections
1530 reflections with *I* > 2σ(*I*)
*R*_int_ = 0.075

#### Refinement

*R*[*F* ^2^ > 2σ(*F* ^2^)] = 0.057
*wR*(*F* ^2^) = 0.079
*S* = 0.92
3147 reflections
155 parameters
H-atom parameters constrained
Δρ_max_ = 0.21 e Å^−3^
Δρ_min_ = −0.22 e Å^−3^

### 

Data collection: *X-AREA* (Stoe & Cie, 2009 ▶); cell refinement: *X-AREA*; data reduction: *X-AREA*; program(s) used to solve structure: *SHELXTL* (Sheldrick, 2008 ▶); program(s) used to refine structure: *SHELXTL*; molecular graphics: *SHELXTL*; software used to prepare material for publication: *SHELXTL* and *PLATON* (Spek, 2009 ▶).

## Supplementary Material

Crystal structure: contains datablock(s) global, I. DOI: 10.1107/S1600536811050902/fj2484sup1.cif

Structure factors: contains datablock(s) I. DOI: 10.1107/S1600536811050902/fj2484Isup2.hkl

Supplementary material file. DOI: 10.1107/S1600536811050902/fj2484Isup3.cml

Additional supplementary materials:  crystallographic information; 3D view; checkCIF report

## Figures and Tables

**Table 1:** Hydrogen-bond geometry (Å, °)

*D*—H⋯*A*	*D*—H	H⋯*A*	*D*⋯*A*	*D*—H⋯*A*
O1—H1⋯N1	0.76	1.97	2.633 (3)	146
N3—H3⋯O1^i^	0.86	2.23	2.919 (3)	138
N3—H3⋯O2^i^	0.86	2.29	3.034 (3)	146

Symmetry code: (i) 
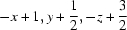
.

**Fig. 1.**
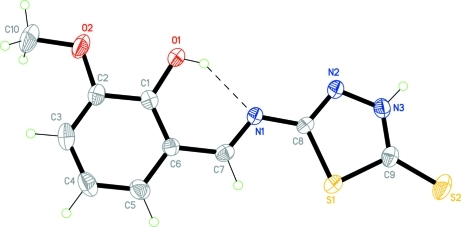
The ORTEP plot of the title compound, showing 40% probability displacement ellipsoids and the atomic numbering. The dashed lines show the intermolecular interaction.

**Fig. 2.**
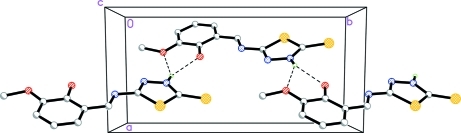
The packing diagram of the title compound viewed down the c-axis showing linkning of molecules through the intermolecular N—H···O intearctions R21(5) ring motifs, forming one-dimensional extended chains along the b-axis. Only the H atoms involved the hydrogen bonds are shown. The dashed lines show the intermolecular interactions.

## supplementary crystallographic information

### Comment

The biological versatility of compounds incorporating a thiadiazole ring is well known (Kumar et al., 1988; Yadav et al., 1989).

The asymmetric unit of the title compound, Fig. 1, comprises a thione-Schiff base ligand. The bond lengths (Allen et al., 1987) and angles are within the normal ranges and are comparable to the related structure (Zhang, 2003; Kargar et al., 2011a,b; Kargar & Kia, 2011).

The dihedral angle between the benzene ring and the five-membered ring is 1.54 (13)°. The intramolecular O—H···N hydrogen bond makes S(6) ring motif (Bernstein et al., 1995). In the crystal packing molecules are linked together through bifurctaed N—H···O hydrogen bonds with R^2^_1_(5) ring motifs (Bernstein et al., 1995), forming one-dimensional extended chains along the b axis. The crystal structure is further stabilized by the intermolecular π-π interactions [[Cg1···Cg2^i^ = 3.767 (3)Å, (i) -x, 1 - y, 1 -z; Cg1···Cg2^ii^ = 3.699 (3)Å, (ii) 1 - x, 1 - y, 1 - z; Cg1 and Cg2 are centroids of S(1)/C(8)/N(2)/N(3)/C(9) and C1–C6 rings, respectively].

### Experimental

The title compound was synthesized by adding 3-methoxy-salicylaldehyde (1 mmol) to a solution of 5-aminothiophene-2-thiol (1 mmol) in ethanol (30 ml). The mixture was refluxed with stirring for half an hour. The resultant solution was filtered. Yellow single crystals of the title compound suitable for *X*-ray structure determination were recrystallized from ethanol by slow evaporation of the solvents at room temperature over several days.

### Refinement

All hydrogen atoms were positioned geometrically with C—H = 0.93-0.96 Å and included in a riding model approximation with U_iso_ (H) = 1.2 or 1.5 U_eq_ (C). A rotating group model was applied to the methyl group.

### Crystal data

**Table:**

C_10_H_9_N_3_O_2_S_2_	*F*(000) = 552
*M**_r_* = 267.32	*D*_x_ = 1.500 Mg m^−^^3^
Monoclinic, *P*2_1_/*c*	Mo *K*α radiation, λ = 0.71069 Å
Hall symbol: -P 2ybc	Cell parameters from 2780 reflections
*a* = 7.432 (5) Å	θ = 2.5–27.4°
*b* = 14.993 (5) Å	µ = 0.44 mm^−^^1^
*c* = 10.853 (5) Å	*T* = 291 K
β = 101.738 (5)°	Block, yellow
*V* = 1184.0 (10) Å^3^	0.25 × 0.21 × 0.11 mm
*Z* = 4	

### Data collection

**Table:**

Stoe IPDS 2T Image Plate diffractometer	3147 independent reflections
Radiation source: fine-focus sealed tube	1530 reflections with *I* > 2σ(*I*)
graphite	*R*_int_ = 0.075
Detector resolution: 0.15 mm pixels mm^-1^	θ_max_ = 29.0°, θ_min_ = 2.4°
ω scans	*h* = −9→10
Absorption correction: multi-scan [*MULABS* (Blessing, 1995) in *PLATON* (Spek, 2009)]	*k* = −20→19
*T*_min_ = 0.898, *T*_max_ = 1.000	*l* = −14→14
9012 measured reflections	

### Refinement

**Table:**

Refinement on *F*^2^	Primary atom site location: structure-invariant direct methods
Least-squares matrix: full	Secondary atom site location: difference Fourier map
*R*[*F*^2^ > 2σ(*F*^2^)] = 0.057	Hydrogen site location: inferred from neighbouring sites
*wR*(*F*^2^) = 0.079	H-atom parameters constrained
*S* = 0.92	*w* = 1/[σ^2^(*F*_o_^2^) + (0.0235*P*)^2^] where *P* = (*F*_o_^2^ + 2*F*_c_^2^)/3
3147 reflections	(Δ/σ)_max_ = 0.001
155 parameters	Δρ_max_ = 0.21 e Å^−^^3^
0 restraints	Δρ_min_ = −0.22 e Å^−^^3^

### Special details

**Table:**

Geometry. All esds (except the esd in the dihedral angle between two l.s. planes) are estimated using the full covariance matrix. The cell esds are taken into account individually in the estimation of esds in distances, angles and torsion angles; correlations between esds in cell parameters are only used when they are defined by crystal symmetry. An approximate (isotropic) treatment of cell esds is used for estimating esds involving l.s. planes.
Refinement. Refinement of F^2^ against ALL reflections. The weighted R-factor wR and goodness of fit S are based on F^2^, conventional R-factors R are based on F, with F set to zero for negative F^2^. The threshold expression of F^2^ > 2sigma(F^2^) is used only for calculating R-factors(gt) etc. and is not relevant to the choice of reflections for refinement. R-factors based on F^2^ are statistically about twice as large as those based on F, and R- factors based on ALL data will be even larger.

### Fractional atomic coordinates and isotropic or equivalent isotropic displacement parameters (Å^2^)

**Table:**

	*x*	*y*	*z*	*U*_iso_*/*U*_eq_	
S1	0.21750 (10)	0.70390 (4)	0.52967 (6)	0.04780 (19)	
S2	0.26803 (11)	0.88098 (5)	0.66246 (8)	0.0669 (3)	
O1	0.3794 (2)	0.35277 (10)	0.59426 (14)	0.0493 (5)	
H1	0.3815	0.4009	0.6167	0.074*	
O2	0.3399 (3)	0.19524 (12)	0.50192 (17)	0.0610 (5)	
N1	0.3081 (3)	0.52471 (12)	0.57179 (17)	0.0366 (5)	
N2	0.4094 (3)	0.63027 (13)	0.72724 (18)	0.0436 (5)	
N3	0.3970 (3)	0.72004 (13)	0.74725 (18)	0.0471 (6)	
H3	0.4552	0.7361	0.8202	0.056*	
C1	0.2778 (3)	0.34679 (16)	0.4758 (2)	0.0380 (6)	
C2	0.2551 (4)	0.26105 (18)	0.4235 (2)	0.0437 (7)	
C3	0.1578 (4)	0.2506 (2)	0.3031 (3)	0.0561 (8)	
H3A	0.1432	0.1940	0.2674	0.067*	
C4	0.0809 (4)	0.3235 (2)	0.2342 (3)	0.0604 (8)	
H4A	0.0156	0.3155	0.1523	0.073*	
C5	0.0996 (4)	0.40713 (18)	0.2846 (2)	0.0528 (7)	
H5A	0.0462	0.4556	0.2375	0.063*	
C6	0.1990 (3)	0.41983 (17)	0.4071 (2)	0.0365 (6)	
C7	0.2189 (3)	0.50812 (16)	0.4597 (2)	0.0395 (6)	
H7A	0.1647	0.5555	0.4103	0.047*	
C8	0.3194 (3)	0.61176 (16)	0.6153 (2)	0.0351 (6)	
C9	0.3028 (4)	0.77222 (16)	0.6575 (2)	0.0438 (6)	
C10	0.2996 (5)	0.10417 (18)	0.4652 (3)	0.0769 (10)	
H10A	0.3550	0.0653	0.5326	0.115*	
H10B	0.3481	0.0910	0.3917	0.115*	
H10C	0.1690	0.0953	0.4468	0.115*	

### Atomic displacement parameters (Å^2^)

**Table:**

	*U*^11^	*U*^22^	*U*^33^	*U*^12^	*U*^13^	*U*^23^
S1	0.0536 (5)	0.0348 (4)	0.0529 (4)	0.0065 (3)	0.0058 (3)	0.0043 (3)
S2	0.0708 (6)	0.0335 (4)	0.0980 (6)	0.0065 (4)	0.0208 (5)	−0.0043 (4)
O1	0.0629 (13)	0.0321 (10)	0.0487 (11)	0.0050 (9)	0.0013 (9)	0.0003 (7)
O2	0.0684 (14)	0.0303 (11)	0.0841 (14)	0.0019 (10)	0.0148 (11)	−0.0055 (9)
N1	0.0394 (13)	0.0303 (12)	0.0397 (12)	0.0015 (10)	0.0071 (10)	−0.0017 (9)
N2	0.0455 (14)	0.0333 (13)	0.0497 (13)	0.0026 (10)	0.0041 (11)	−0.0036 (10)
N3	0.0482 (14)	0.0407 (14)	0.0499 (13)	−0.0017 (11)	0.0044 (11)	−0.0094 (11)
C1	0.0352 (15)	0.0415 (15)	0.0389 (13)	−0.0042 (12)	0.0112 (12)	−0.0040 (12)
C2	0.0428 (17)	0.0374 (16)	0.0540 (17)	−0.0030 (13)	0.0175 (14)	−0.0048 (13)
C3	0.062 (2)	0.0465 (19)	0.0655 (19)	−0.0168 (15)	0.0269 (16)	−0.0203 (15)
C4	0.067 (2)	0.064 (2)	0.0484 (16)	−0.0238 (18)	0.0068 (15)	−0.0136 (16)
C5	0.0549 (19)	0.0518 (19)	0.0478 (17)	−0.0114 (15)	0.0013 (14)	0.0021 (13)
C6	0.0338 (15)	0.0358 (15)	0.0399 (14)	−0.0044 (12)	0.0074 (12)	0.0008 (11)
C7	0.0374 (16)	0.0366 (16)	0.0449 (15)	0.0006 (12)	0.0094 (13)	0.0077 (12)
C8	0.0344 (14)	0.0309 (14)	0.0414 (14)	0.0033 (11)	0.0108 (12)	0.0054 (11)
C9	0.0390 (15)	0.0376 (15)	0.0578 (16)	−0.0019 (13)	0.0167 (13)	−0.0025 (13)
C10	0.083 (3)	0.0348 (18)	0.115 (3)	−0.0048 (17)	0.026 (2)	−0.0109 (18)

### Geometric parameters (Å, °)

**Table:**

S1—C9	1.737 (3)	C1—C2	1.401 (3)
S1—C8	1.749 (2)	C2—C3	1.369 (3)
S2—C9	1.654 (3)	C3—C4	1.381 (4)
O1—C1	1.355 (3)	C3—H3A	0.9300
O1—H1	0.7607	C4—C5	1.364 (3)
O2—C2	1.370 (3)	C4—H4A	0.9300
O2—C10	1.437 (3)	C5—C6	1.397 (3)
N1—C7	1.286 (3)	C5—H5A	0.9300
N1—C8	1.385 (3)	C6—C7	1.437 (3)
N2—C8	1.292 (3)	C7—H7A	0.9300
N2—N3	1.369 (3)	C10—H10A	0.9600
N3—C9	1.332 (3)	C10—H10B	0.9600
N3—H3	0.8561	C10—H10C	0.9600
C1—C6	1.386 (3)		
			
C9—S1—C8	89.62 (13)	C4—C5—H5A	120.1
C1—O1—H1	109.8	C6—C5—H5A	120.1
C2—O2—C10	118.0 (2)	C1—C6—C5	119.2 (2)
C7—N1—C8	119.3 (2)	C1—C6—C7	121.1 (2)
C8—N2—N3	108.7 (2)	C5—C6—C7	119.7 (2)
C9—N3—N2	120.2 (2)	N1—C7—C6	123.0 (2)
C9—N3—H3	127.1	N1—C7—H7A	118.5
N2—N3—H3	112.7	C6—C7—H7A	118.5
O1—C1—C6	123.5 (2)	N2—C8—N1	120.5 (2)
O1—C1—C2	116.3 (2)	N2—C8—S1	114.54 (18)
C6—C1—C2	120.3 (2)	N1—C8—S1	125.01 (18)
C3—C2—O2	126.7 (3)	N3—C9—S2	128.1 (2)
C3—C2—C1	119.3 (3)	N3—C9—S1	106.85 (18)
O2—C2—C1	114.0 (2)	S2—C9—S1	125.01 (17)
C2—C3—C4	120.4 (3)	O2—C10—H10A	109.5
C2—C3—H3A	119.8	O2—C10—H10B	109.5
C4—C3—H3A	119.8	H10A—C10—H10B	109.5
C5—C4—C3	120.9 (3)	O2—C10—H10C	109.5
C5—C4—H4A	119.5	H10A—C10—H10C	109.5
C3—C4—H4A	119.5	H10B—C10—H10C	109.5
C4—C5—C6	119.9 (3)		
			
C8—N2—N3—C9	0.0 (3)	C4—C5—C6—C1	0.1 (4)
C10—O2—C2—C3	−11.4 (4)	C4—C5—C6—C7	179.8 (2)
C10—O2—C2—C1	169.9 (2)	C8—N1—C7—C6	−179.6 (2)
O1—C1—C2—C3	−178.4 (2)	C1—C6—C7—N1	−0.5 (4)
C6—C1—C2—C3	1.3 (4)	C5—C6—C7—N1	179.8 (2)
O1—C1—C2—O2	0.4 (3)	N3—N2—C8—N1	−179.1 (2)
C6—C1—C2—O2	−179.9 (2)	N3—N2—C8—S1	0.5 (2)
O2—C2—C3—C4	−179.3 (3)	C7—N1—C8—N2	−179.5 (2)
C1—C2—C3—C4	−0.7 (4)	C7—N1—C8—S1	1.0 (3)
C2—C3—C4—C5	−0.3 (4)	C9—S1—C8—N2	−0.61 (19)
C3—C4—C5—C6	0.6 (4)	C9—S1—C8—N1	178.9 (2)
O1—C1—C6—C5	178.7 (2)	N2—N3—C9—S2	179.02 (18)
C2—C1—C6—C5	−1.0 (4)	N2—N3—C9—S1	−0.4 (3)
O1—C1—C6—C7	−1.0 (4)	C8—S1—C9—N3	0.53 (18)
C2—C1—C6—C7	179.3 (2)	C8—S1—C9—S2	−178.93 (18)

### Hydrogen-bond geometry (Å, °)

**Table:**

*D*—H···*A*	*D*—H	H···*A*	*D*···*A*	*D*—H···*A*
O1—H1···N1	0.76	1.97	2.633 (3)	146.
N3—H3···O1^i^	0.86	2.23	2.919 (3)	138.
N3—H3···O2^i^	0.86	2.29	3.034 (3)	146.

Symmetry codes: (i) −*x*+1, *y*+1/2, −*z*+3/2.

## References

[bb1] Allen, F. H., Kennard, O., Watson, D. G., Brammer, L., Orpen, A. G. & Taylor, R. (1987). *J. Chem. Soc. Perkin Trans. 2*, pp. S1–19.

[bb2] Bernstein, J., Davis, R. E., Shimoni, L. & Chang, N.-L. (1995). *Angew. Chem. Int. Ed. Engl.* **34**, 1555–1573.

[bb3] Blessing, R. H. (1995). *Acta Cryst.* A**51**, 33–38. 10.1107/s0108767394005726 7702794

[bb5] Kargar, H. & Kia, R. (2011). *Acta Cryst.* E**67**, o3437. 10.1107/S1600536811049877 PMC3239070 22199918

[bb6] Kargar, H., Kia, R. & Tahir, M. N. (2011*a*). *Acta Cryst.* E**67**, o3311. 10.1107/S1600536811047362 PMC3238963 22199812

[bb7] Kargar, H., Kia, R. & Tahir, M. N. (2011*b*). *Acta Cryst.* E**67**, o3436. 10.1107/S1600536811049920 PMC3239069 22199917

[bb8] Kumar, R., Giri S. & Nizamuddin (1988). *J. Indian Chem. Soc.* **65**, 572–573.

[bb9] Sheldrick, G. M. (2008). *Acta Cryst.* A**64**, 112–122. 10.1107/S0108767307043930 18156677

[bb10] Spek, A. L. (2009). *Acta Cryst.* D**65**, 148–155. 10.1107/S090744490804362X PMC2631630 19171970

[bb11] Stoe & Cie (2009). *X-AREA* Stoe & Cie, Darmstadt, Germany.

[bb12] Yadav, L. D. S., Shukla, K. N. & Singh, H. (1989). *Indian J. Chem. Sect B*, **28**, 78–80.

[bb13] Zhang, Y.-X. (2003). *Acta Cryst.* E**59**, o581–o582.

